# Risk factors associated with mortality in patients infected with influenza A/H1N1 in Mexico

**DOI:** 10.1186/s13104-015-1349-8

**Published:** 2015-09-11

**Authors:** Luis Alberto Mata-Marín, José Antonio Mata-Marín, Velasco Claudia Vásquez-Mota, Carla Ileana Arroyo-Anduiza, Jesús Enrique Gaytán-Martínez, Bulmaro Manjarrez-Téllez, Luis Alberto Ochoa-Carrera, Jorge Luis Sandoval-Ramírez

**Affiliations:** Cardiology and Angiology Department, Herzzentrum am Klinikum Links der Weser, Senator-Weßling-Straße 1, 28277 Bremen, Germany; Infectious Diseases Department, Hospital de Infectología, “La Raza” National Medical Center, IMSS, Mexico City, Mexico; Clinical Pathology Department, Hospital General, “La Raza” National Medical Center, IMSS, Mexico City, Mexico; Central Blood Bank, “La Raza” National Medical Center, IMSS, Mexico City, Mexico; Immunology and Infectious Diseases Research Unit, “La Raza” National Medical Center, IMSS, Mexico City, Mexico

**Keywords:** Epidemic, Influenza, Influenza A/H1N1, Mortality, Risk factors

## Abstract

**Background:**

Influenza virus pandemics vary dramatically in their severity and mortality. Thus, it is very important to identify populations with high risks of developing severe illness to reduce mortality in future pandemics. The purpose was to determine the mortality-associated risk factors in hospitalized Mexican patients infected with influenza A/H1N1.

**Results:**

The risk factors associated with mortality were: male sex [odds ratio (OR) = 5.25, confidence interval (CI) = 1.22–28.95], medical attention delayed >3 days (OR = 9.9, CI = 1.51–64.52), anti-flu therapy delayed >3 days (OR = 10.0, CI = 1.07–93.43), admission to intensive care unit (ICU) (OR = 9.9, CI = 1.51–64.52) and creatinine levels >1.0 mg/dL when admitted to hospital (OR = 11.2, CI = 1.05–120.32). After adjusting for the effects of potentially confounding variables in a logistic regression model, delayed medical attention (OR = 13.91, CI = 1.09–41.42, *p* = 0.044) and ICU hospitalization (OR = 11.02, CI = 1.59–76.25, *p* = 0.015) were the only predictors of mortality.

**Conclusion:**

Early medical attention is essential for reducing the mortality risk in patients with influenza A/H1N1, while a requirement for ICU management increases the risk.

## Background

Pandemic influenza A (H1N1) virus infection varies in its severity. However, little is known about the infectivity and transmissibility of the 2009 H1N1 pandemic influenza (pH1N1) virus outside Mexico. Pneumonia was reported to be the major cause of hospitalization and death in confirmed cases of H1N1 [1]. Delayed antiviral treatment, severe hypoxemia and multisystem organ failure have been reported to be negative prognostic factors in critically ill patients with influenza, while early diagnosis, assessment of high risk and the analysis of epidemic pneumonia may be useful for assessing the risk of this virus and the possibility of its control [2].

Active surveillance of severe pneumonia was initiated at the Instituto Mexicano del Seguro Social (IMSS) after the first epidemiological alert during April 2009 when influenza was not expected to reach epidemic levels. In total, 117,626 influenza-like illness (ILI) cases were reported by IMSS between April and December 2009, of which 36,044 were laboratory-tested (30.6 %) and 27,440 (23.3 %) were confirmed as pandemic influenza A/H1N1. A total of 1370 ILI deaths (3.6 per 100,000) were reported to the surveillance system, of which 585 (1.5 per 100,000) were confirmed as A/H1N1 virus infections [3–6]. Of 6945 cases reported by IMSS as novel A/H1N1 infections, <1 % died and 7 % were hospitalized [3–6]. Another study was conducted in six hospitals between March and June during 2009, which showed that 58/899 hospitalized patients developed critical illness [4, 5].

The clinical, virological and immunological parameters associated with the clinical severity of this virus have been explored in recent studies [6, 7]. The influenza virus is primarily a respiratory pathogen but the clinical manifestations in severe infections are not always restricted to the respiratory tract and they more closely resemble those of a systemic infection. In some countries, 10–30 % of patients with H1N1 infection required admission to intensive care units (ICUs) [8].

Most previous studies of the 2009 H1N1 outbreak in Mexico focused on critically ill patients [9–11]. The H1N1 virus pandemic caused significant morbidity and mortality in certain demographic groups, such as the very old, pregnant women and obese patients [12].

The aim of this study was to identify clinically useful predictors of mortality and the disease severity of H1N1 pneumonia.

## Methods

The study was conducted in accordance with the ethics principles of the Declaration of Helsinki (Helsinki, Finland). Written informed consent was obtained from each participant. The study was approved by the Local Committee for Health Research 35021 Hospital de Infectologia “La Raza” National Medical Center, with corporate registration number R-2010-35021-5.

## Surveillance and data collection

ILI was defined as a combination of cough, headache and fever (except in persons aged over 65 years) with one or more of the following symptoms: sore throat, rhinorrhoea, arthralgia, myalgia, prostration, thoracic pain, abdominal pain, nasal congestion, diarrhoea and irritability [3].

### Participants

A case–control study was designed to investigate the factors associated with unfavourable prognosis (mortality) among in-patients with documented influenza A/H1N1 infection. The participants attended the Hospital de Infectología, National Medical Center “La Raza”, in Mexico City between April and November 2009. The cases were patients who died during hospitalization whereas the controls were discharged after their improvement. The patients included were aged >18 years and were confirmed as A/H1N1-positive by reverse transcriptase PCR. We defined the patients with severe disease as those who died with confirmed influenza A/H1N1 infection, or those who were admitted to the ICU and/or were intubated for severe respiratory symptoms associated with influenza A/H1N1. A total of 33 patients met the inclusion criteria in this medical centre.

### Sample collection

We obtained ILI surveillance data and the results of laboratory-tested samples for all individuals who sought hospital medical care for ILI symptoms between April and November during 2009. Nasopharyngeal swabs and/or bronchoalveolar lavage samples were sent to the diagnostic reference laboratory with standardized questionnaires, including demographic and epidemiological/clinical information. The swab samples and specimens were homogenized in virus transport medium and kept on ice before diagnosis. The data collected included, general attributes, medical history, symptoms, records of respiratory evolution and routine laboratory tests. Each suspected case of ILI was laboratory-confirmed as negative or positive for pandemic influenza A/H1N1. The diagnosis of influenza was invariably provided within 8–24 h after the receipt of the sample. Swabs were tested for A/H1N1 influenza virus by real-time PCR at the Instituto de Diagnóstico y Referencia Epidemiológica (InDRE), after which point samples were analysed by “La Raza”, which is an IMSS laboratory certified by InDRE [3]. To perform a rapid diagnosis, specific primers and probes were used to detect influenza A/H1N1. The quantitative real-time PCR primers were based on the conserved matrix protein gene, according to World Health Organization (WHO) recommendations. Further analyses and confirmation were carried out according to Center for Disease Control and Prevention and WHO guidelines.

### Statistical analysis

The results were compared using the Mann–Whitney *U-*test and *p* < 0.05 was considered sufficient to merit investigation as a factor associated with unfavourable prognosis (mortality) among in-patients with documented influenza A/H1N1 infection.

A logistic regression model was used to analyse the associations between the variables of interest. The results were expressed as odds ratios (ORs), and the 95 % confidence intervals (CIs) were calculated for each predictive factor and mortality risk. The statistical analyses were performed using SPSS 17.0 for Windows (SPSS, Inc., Chicago, IL, USA).

## Results

### Patient characteristics

In total, 33 patients with influenza A/H1N1 infection were confirmed by quantitative real-time PCR, of whom 11 were cases (patients who died) and 22 were controls (patients who were discharged after improvement). Twelve of the patients (38 %) were men: seven (63 %) in the case group and five (25 %) in the control group. The medians of the subject age inter-quartile ranges (IQRs) were 29 years (IQR = 25–46) and 34 years (IQR = 22–44), respectively (*p* = 0.685). Ten (90 %) cases and four (13 %) controls were hospitalized in the ICU (*p* < 0.001). The cases experienced first medical visit delays of 3 days (IQR = 2–7) vs 1 day (IQR = 1–3), (*p* = 0.043) and oseltamivir treatment initiation delays of 8 days (IQR = 6–10) vs 3 days (IQR = 2–7) (*p* = 0.038) (Table 1).

**Table 1:** Baseline characteristics of patients with H1N1 infections

Characteristics	Cases n = 11 (%)	Controls n = 22 (%)	*p* value
Male	7 (63.6)	5 (22.7)	0.035*
Age	29 (IQR = 25–46)	34 (IQR = 22–44)	0.685
Lack of flu vaccination in previous year	11 (100)	15 (78.9)	0.674
Smoker	5 (45.5)	10 (45.5)	0.999
Overweight or obese	7 (63.6)	13 (59.1)	0.604
Chronic disease diagnosis^a^	5 (45.5)	4 (18.2)	0.097
First medical attention after onset of flu symptoms (days)	3 (IQR = 2–7)	1 (IQR = 1–3)	0.043*
Treatment initiation with oseltamivir after onset of flu symptoms (days)	8 (IQR = 6–10)	3 (IQR = 2–7)	0.038*
Intensive care unit hospitalization	10 (90.9)	3 (13.6)	0.001*
Received oseltamivir	10 (90.9)	20 (90.1)	0.999

The data are expressed as percentages (%) or averages.* p* value according to Student’s t-test, *χ* ^2^ test or Fisher’s exact test. Median (IQR = 25–75)

*IQR* inter-quartile range

* p < 0.05

^a^Type 2 diabetes, hypertension, asthma or HIV infection

### Risk factors associated with mortality

The risk factors associated with mortality were: male sex (OR = 5.25, CI = 1.22–28.95), ICU hospitalization (OR = 9.9, CI = 1.51–64.52), medical attention delayed >3 days (OR = 6.41, CI = 1.18–34.61), initiation of anti-flu therapy (oseltamivir) delayed >3 days (OR = 10.0, CI = 1.07–93.43) and serum creatinine >1.0 mg/dL (OR = 11.2, CI = 1.05–120.32, *p* = 0.004) (Table 2). After adjusting for the effects of potentially confounding variables in a logistic regression model, we found that delayed medical attention (OR = 11.02, *p* = 0.015) and ICU hospitalization (OR = 13.91, *p* = 0.044) were the only predictors of mortality (Table 3).

**Table 2:** Risk factors associated with mortality in patients with influenza A/H1N1 based on a bivariate analysis

Risk factor	Bivariate analysis	
	OR (95 % CI)	*p* value
Male	5.25 (1.22–28.95)	0.004*
Lack of flu vaccination in previous year	11.12 (0.57–215.3)	0.057
Recent or past smoker	0.88 (0.18–4.38)	0.885
Overweight or obese	0.62 (0.09–3.91)	0.609
Chronic disease diagnosis†	3.12 (0.62–15.79)	0.160
Intensive care unit hospitalization	9.9 (1.51–64.52)	<0.001*
Medical received attention after >3 days	6.41 (1.18–34.61)	0.018*
Anti-flu treatment after >3 days	10.0 (1.07–93.43)	0.039*
Serum creatinine >1.0 mg/dL	11.2 (1.05–120.32)	0.004*

*OR* odds ratio, *CI* confidence interval

* Significant difference at *p* < 0.05

**Table 3:** Risk factors associated with mortality in patients with influenza A/H1N1 based on a multivariate analysis

Risk factor	Multivariate analysis	
	OR (95 % CI)	*p* value
First medical attention received after >3 days	11.02 (1.59–76.25)	0.015*
Male	2.73 (0.52–14.28)	0.233
Age	1.02 (0.95–1.01)	0.549
Recent or past smoker	1.19 (0.16–8.92)	0.867
Chronic disease diagnosis	4.91 (0.58–41.42)	0.144
Intensive care unit hospitalization	13.91 (1.09–41.42)	0.044*

*OR* odds ratio, *CI* confidence interval

* Significant difference at *p* < 0.05

## Discussion

During early April 2009, medical care units were alerted that the number of seasonal influenza cases had not decreased to expected levels and it was later confirmed that a novel influenza A/H1N1 virus caused the outbreak. The baseline estimate of the disease outbreak was that approximately 375,000 Mexicans had novel H1N1 influenza infections with onset of symptoms by approximately 30 April, 2009 [13]. The increasing levels of hospitalized patients led to the design of a case–control study of hospitalized patients at the Hospital de Infectología, National Medical Center “La Raza”. This study elucidated the risk factors associated with influenza A/H1N1 mortality. We found that delayed medical attention and ICU hospitalization were predictive factors associated with mortality in our population.

Most of the cases who died required ICU admission because of the disease severity. Similar data were reported in four previous studies. Patients with influenza H1N1 were evaluated by Dominguez Cherit et al. [14] in Mexico and by Kumar et al. [15] in Canada. In Mexico, Echeverría-Zuno et al. and Louie et al. reported that all of the patients who died were admitted to ICU services. However, the patients differed with respect to the need for endotracheal intubation because there was no need for this procedure in Canada whereas 2/24 patients required intubation in Mexico [16, 17]. One of our patients died and was not ICU hospitalized because the patient arrived at our hospital in a very bad condition and died in the emergency room.

Of the 11 cases evaluated, seven were male. A recent study in Canada showed that of 29 deaths, eight were men (27.6 %) and 21 women (72.4 %), although most studies state that male sex is a risk factor for acquiring influenza A/H1N1 infection [15, 18]. A significant result was obtained for elevated serum creatinine (only in patients with confirmed H1N1) but it was not a predictive factor for mortality. Elevated serum creatinine was detected in 11 cases and it was statistically significant [14, 18, 19].

The appropriate and timely use of oseltamivir to treat early influenza symptoms reduces the complications of the disease and the risk of mortality. Similar results have been reported in China, Mexico, Canada and the United States where the use of oseltamivir 48 h after the onset of symptoms reduces the risk of mortality, especially in pregnant women. This situation is acknowledged by the WHO guidelines, which indicate that early initiation of oseltamivir for suspected influenza cases in pregnant women decreases the mortality risk whereas delayed medical attention increases the mortality rate [16–21]. The beneficial early therapy with oseltamivir could be not useful in resistant virus like described in another studies and the intravenous medicaments have been used in patients with seasonal influenza and against Influenza A/H1N1 there are just in vitro beneficial results, randomized controlled trials there are not still made however [21].

This pandemic virus, suggest that we have to be prepared against agents of emerging infectious diseases, in future pandemics, we have to act faster to prevent dissemination. The influenza virus is highly unpredictable, and we should be ready for another pandemia. The challenges in successful vaccination against influenza lie in their variable efficacy in different age populations, the antigenic variability of the circulating virus, and the production and manufacturing limitations to ensure safe, timely, and adequate supply of vaccine. Future improvement will require novel approaches to both vaccine formulation and manufacturing processes. The actually studies to find a universal vaccine targeting all influenza virus with the expressed HA antigens are still under investigation, but until then we have to be prepared just with the antiviral weapons that by now we have and to our patients a early therapy offer to fast as possible [22].

The sample size was a limiting factor. The study was initiated during the second outbreak of the epidemic and there were a lower number of cases, which affected the number of patients available for collecting data, for that, this results should be taken carefully. In addition, most of the referred patients had already initiated antiviral therapy. However, the data were collected directly from the patients or using a questionnaire, and we followed the course of each patient until their death or improvement, using confirmatory tests and/or clinical checks.

## Conclusions

In conclusion, the early detection of the signs and symptoms of this disease, immediate medical attention, antiviral therapy and respiratory therapy slow the progression of influenza A/H1N1 and reduce the risk of mortality. The patients with a requirement for ICU management have an increased risk of mortality.

## Abbreviations

IMSS: Instituto Mexicano del Seguro Social
ILI: influenza like illness
ICU: intensive care unit
PCR: polymerase chain reaction
InDRE: Instituto de Diagnóstico y Referencia Epidemiológica
WHO: Wolrd Health Organisation
OR: odds ratio
CI: confidence interval
IQR: inter-quartile-ranges
